# A second monoclinic polymorph of methyl 4-hydroxy­benzoate

**DOI:** 10.1107/S1600536808017327

**Published:** 2008-06-13

**Authors:** Hoong-Kun Fun, Samuel Robinson Jebas

**Affiliations:** a X-ray Crystallography Unit, School of Physics, Universiti Sains Malaysia, 11800 USM, Penang, Malaysia

## Abstract

A second monoclinic polymorph of methyl 4-hydroxy­benzoate, C_8_H_8_O_3_, is reported. The unit-cell dimensions are different from those of the previously reported monoclinic form [Vujovic & Nassimbeni (2006 ▶). *Cryst. Growth Des.* **6**, 1595–1597]. The asymmetric unit contains three crystallographically independent mol­ecules, as observed in the previous form. The crystal structure is stabilized by inter­molecular O—H⋯O and C—H⋯O hydrogen bonds and C—H⋯π inter­actions, which link the mol­ecules into a three-dimensional network.

## Related literature

For the other monoclinic polymorph of methyl 4-hydroxy­benzoate, see: Lin (1983 ▶); Vujovic & Nassimbeni (2006 ▶).
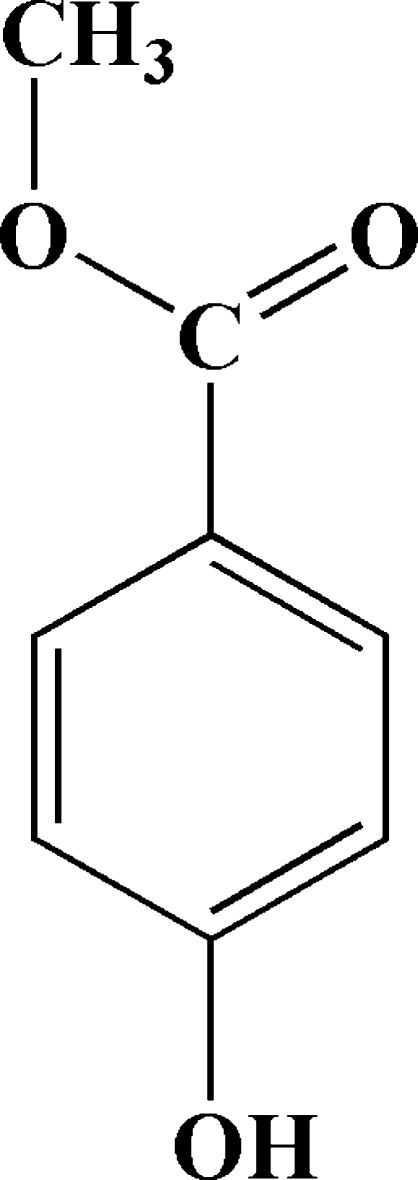


## Experimental

### 

#### Crystal data

C_8_H_8_O_3_
*M* *_r_* = 152.14
Monoclinic, 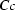

*a* = 12.9708 (4) Å
*b* = 17.2485 (7) Å
*c* = 10.8428 (3) Å
β = 119.260 (1)°
*V* = 2116.32 (12) Å^3^
*Z* = 12
Mo *K*α radiation
μ = 0.11 mm^−1^
*T* = 100.0 (1) K
0.29 × 0.27 × 0.19 mm

#### Data collection

Bruker SMART APEXII CCD area-detector diffractometer
Absorption correction: multi-scan (*SADABS*; Bruker, 2005 ▶) *T*_min_ = 0.969, *T*_max_ = 0.979
25224 measured reflections
3278 independent reflections
2705 reflections with *I* > 2σ(*I*)
*R*_int_ = 0.047

#### Refinement

*R*[*F* ^2^ > 2σ(*F* ^2^)] = 0.043
*wR*(*F* ^2^) = 0.116
*S* = 1.05
3278 reflections
301 parameters
2 restraints
H-atom parameters constrained
Δρ_max_ = 0.40 e Å^−3^
Δρ_min_ = −0.24 e Å^−3^

### 

Data collection: *APEX2* (Bruker, 2005 ▶); cell refinement: *APEX2*; data reduction: *SAINT* (Bruker, 2005 ▶); program(s) used to solve structure: *SHELXTL* (Sheldrick, 2008 ▶); program(s) used to refine structure: *SHELXTL*; molecular graphics: *SHELXTL*; software used to prepare material for publication: *SHELXTL* and *PLATON* (Spek, 2003 ▶).

## Supplementary Material

Crystal structure: contains datablocks global, I. DOI: 10.1107/S1600536808017327/ci2607sup1.cif

Structure factors: contains datablocks I. DOI: 10.1107/S1600536808017327/ci2607Isup2.hkl

Additional supplementary materials:  crystallographic information; 3D view; checkCIF report

## Figures and Tables

**Table 1:** Hydrogen-bond geometry (Å, °)

*D*—H⋯*A*	*D*—H	H⋯*A*	*D*⋯*A*	*D*—H⋯*A*
O1*A*—H1*A*⋯O2*A*^i^	0.82	1.96	2.770 (2)	168
O1*B*—H1*B*⋯O3*C*^ii^	0.82	1.93	2.729 (2)	167
O1*C*—H1*C*⋯O2*B*	0.82	1.92	2.729 (2)	167
C6*A*—H6*A*⋯O2*C*	0.93	2.58	3.343 (3)	140
C8*C*—H8*C*1⋯*Cg*1^i^	0.96	2.76	3.539 (3)	139
C8*C*—H8*C*3⋯*Cg*2	0.96	2.70	3.442 (3)	134
C8*A*—H8*A*1⋯*Cg*3^iii^	0.96	2.68	3.515 (3)	145
C8*B*—H8*B*3⋯*Cg*3^iv^	0.96	2.78	3.655 (4)	151

Symmetry codes: (i) 
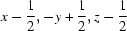
; (ii) 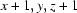; (iii) 
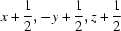
; (iv) 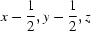. *Cg*1, *Cg*2 and *Cg*3 are the centroids of the C1*A*–C6*A*, C1*B*–C6*B* and C1*C*–C6*C* rings, respectively.

**Fig. 1.**
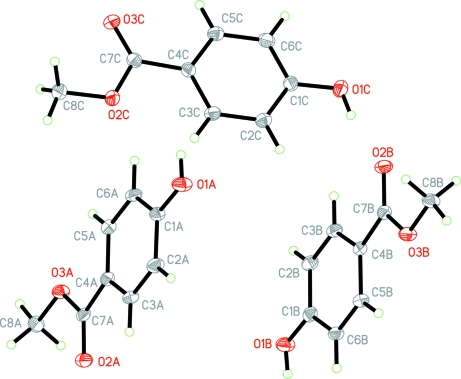
The asymmetric unit of the title compound, showing 50% probability displacement ellipsoids and the atom-numbering scheme.

**Fig. 2.**
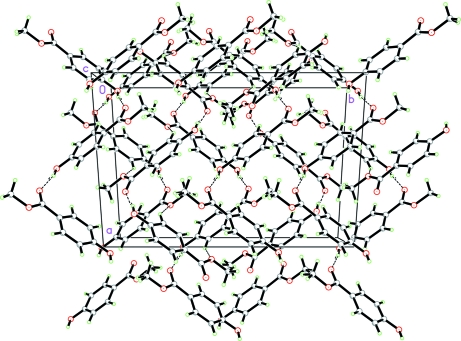
The crystal packing of the title compound, viewed along the c axis. Hydrogen bonds are shown as dashed lines.

## supplementary crystallographic information

### Comment

The crystal structure of the title compound at room temperature and at 113 K has been reported previously (Lin, 1983; Vujovic & Nassimbeni, 2006). We report here the structure of a second monoclinic polymorph of the title compound which was elucidated at 100.0 (1) K.

The compound crystallizes in the space group *Cc* with three independent molecules in the asymmetric unit, similar to the first monoclinic polymorph (Vujovic & Nassimbeni, 2006). However, the cell parameters of the present monoclinic polymorph differ significantly from the previous polymorph [a = 13.006 (3) Å, b = 17.261 (4) Å, c = 12.209 (2) Å and β = 129.12 (3)°]. The corresponding bond lengths and angles of the three independent molecules agree with each other and also with those in the other monoclinic polymorph (Vujovic & Nassimbeni, 2006). Each of the independent molecules are planar. The dihedral angles formed by the C1A-C6A plane with the C1B-C6B and C1C-C6C planes are 2.9 (1)° and 71.2 (1)°, respectively. In the first monoclinic polymorph (Vujovic & Nassimbeni, 2006) these angles are 2.9 (1) and 1.4 (1)°.

In the asymmetric unit, the independent molecules are linked via O—H···O and C—H···O hydrogen bonds. The crystal packing  is stabilized by intermolecular O—H···O and C—H···O hydrogen bonds and C—H···π interactions which link the molecules into a three-dimensional network (Fig.2).

### Experimental

Methyl 4-hydroxybenzoate was purchased from Aldrich. Single crystals were obtained by slow evaporation of an ethanol solution.

### Refinement

H atoms were positioned geometrically [C-H = 0.93-0.96 Å and O-H = 0.82 Å] and refined using a riding model, with *U*_iso_(H) = 1.2*U*_eq_(C). In the absence of significant anomalous scattering, 3197 Friedel pairs were merged prior to the final refinement.

### Crystal data

**Table:**

C_8_H_8_O_3_	*F*_000_ = 960
*M**_r_* = 152.14	*D*_x_ = 1.433 Mg m^−^^3^
Monoclinic, *C**c*	Mo *K*α radiation λ = 0.71073 Å
Hall symbol: C -2yc	Cell parameters from 6163 reflections
*a* = 12.9708 (4) Å	θ = 2.3–28.8º
*b* = 17.2485 (7) Å	µ = 0.11 mm^−^^1^
*c* = 10.8428 (3) Å	*T* = 100.0 (1) K
β = 119.260 (1)º	Block, purple
*V* = 2116.32 (12) Å^3^	0.29 × 0.27 × 0.19 mm
*Z* = 12	

### Data collection

**Table:**

Bruker SMART APEXII CCD area-detector diffractometer	3278 independent reflections
Radiation source: fine-focus sealed tube	2705 reflections with *I* > 2σ(*I*)
Monochromator: graphite	*R*_int_ = 0.047
*T* = 100.0(1) K	θ_max_ = 30.6º
φ and ω scans	θ_min_ = 2.2º
Absorption correction: multi-scan(SADABS; Bruker, 2005)	*h* = −18→18
*T*_min_ = 0.969, *T*_max_ = 0.979	*k* = −24→24
25224 measured reflections	*l* = −15→15

### Refinement

**Table:**

Refinement on *F*^2^	Secondary atom site location: difference Fourier map
Least-squares matrix: full	Hydrogen site location: inferred from neighbouring sites
*R*[*F*^2^ > 2σ(*F*^2^)] = 0.043	H-atom parameters constrained
*wR*(*F*^2^) = 0.116	*w* = 1/[σ^2^(*F*_o_^2^) + (0.0693*P*)^2^ + 0.2705*P*] where *P* = (*F*_o_^2^ + 2*F*_c_^2^)/3
*S* = 1.06	(Δ/σ)_max_ = 0.001
3278 reflections	Δρ_max_ = 0.40 e Å^−^^3^
301 parameters	Δρ_min_ = −0.24 e Å^−^^3^
2 restraints	Extinction correction: none
Primary atom site location: structure-invariant direct methods	

### Special details

**Table:**

Experimental. The data was collected with the Oxford Cyrosystem Cobra low-temperature attachment.
Geometry. All e.s.d.'s (except the e.s.d. in the dihedral angle between two l.s. planes) are estimated using the full covariance matrix. The cell e.s.d.'s are taken into account individually in the estimation of e.s.d.'s in distances, angles and torsion angles; correlations between e.s.d.'s in cell parameters are only used when they are defined by crystal symmetry. An approximate (isotropic) treatment of cell e.s.d.'s is used for estimating e.s.d.'s involving l.s. planes.
Refinement. Refinement of *F*^2^ against ALL reflections. The weighted *R*-factor *wR* and goodness of fit *S* are based on *F*^2^, conventional *R*-factors *R* are based on *F*, with *F* set to zero for negative *F*^2^. The threshold expression of *F*^2^ > σ(*F*^2^) is used only for calculating *R*-factors(gt) *etc*. and is not relevant to the choice of reflections for refinement. *R*-factors based on *F*^2^ are statistically about twice as large as those based on *F*, and *R*- factors based on ALL data will be even larger.

### Fractional atomic coordinates and isotropic or equivalent isotropic displacement parameters (Å^2^)

**Table:**

	*x*	*y*	*z*	*U*_iso_*/*U*_eq_	
O1A	0.29177 (16)	0.36449 (10)	0.60077 (19)	0.0230 (4)	
H1A	0.2494	0.3725	0.5160	0.034*	
O2A	0.66456 (16)	0.08828 (9)	0.82151 (18)	0.0209 (4)	
O3A	0.57101 (15)	0.06897 (9)	0.58740 (18)	0.0181 (3)	
C1A	0.3604 (2)	0.30138 (13)	0.6194 (3)	0.0171 (5)	
C2A	0.4381 (2)	0.27923 (15)	0.7586 (3)	0.0202 (5)	
H2A	0.4414	0.3072	0.8337	0.024*	
C3A	0.5102 (2)	0.21526 (14)	0.7836 (2)	0.0182 (5)	
H3A	0.5621	0.2005	0.8762	0.022*	
C4A	0.5061 (2)	0.17242 (14)	0.6716 (3)	0.0161 (5)	
C5A	0.4260 (2)	0.19429 (13)	0.5327 (3)	0.0167 (5)	
H5A	0.4209	0.1657	0.4572	0.020*	
C6A	0.3542 (2)	0.25858 (14)	0.5077 (3)	0.0181 (5)	
H6A	0.3014	0.2731	0.4152	0.022*	
C7A	0.58804 (19)	0.10701 (13)	0.7037 (2)	0.0151 (5)	
C8A	0.6476 (2)	0.00343 (13)	0.6093 (3)	0.0198 (5)	
H8A1	0.7280	0.0209	0.6512	0.030*	
H8A2	0.6256	−0.0209	0.5202	0.030*	
H8A3	0.6401	−0.0332	0.6712	0.030*	
O1B	1.02411 (15)	0.30607 (10)	0.65189 (19)	0.0217 (4)	
H1B	1.0678	0.2996	0.7369	0.033*	
O2B	0.65142 (15)	0.58228 (9)	0.42254 (18)	0.0213 (4)	
O3B	0.74271 (14)	0.60350 (9)	0.65507 (17)	0.0192 (3)	
C1B	0.95497 (19)	0.36928 (13)	0.6311 (2)	0.0170 (5)	
C2B	0.8748 (2)	0.38876 (14)	0.4925 (2)	0.0174 (5)	
H2B	0.8700	0.3591	0.4182	0.021*	
C3B	0.8020 (2)	0.45249 (13)	0.4652 (2)	0.0168 (4)	
H3B	0.7476	0.4650	0.3723	0.020*	
C4B	0.8090 (2)	0.49832 (13)	0.5752 (2)	0.0149 (4)	
C5B	0.89218 (19)	0.47907 (13)	0.7148 (2)	0.0166 (4)	
H5B	0.8988	0.5096	0.7890	0.020*	
C6B	0.9648 (2)	0.41474 (13)	0.7432 (3)	0.0166 (4)	
H6B	1.0195	0.4020	0.8359	0.020*	
C7B	0.72729 (19)	0.56418 (13)	0.5416 (2)	0.0161 (4)	
C8B	0.6628 (3)	0.66766 (15)	0.6307 (3)	0.0198 (4)	
H8B1	0.6704	0.7050	0.5699	0.030*	
H8B2	0.6820	0.6916	0.7192	0.030*	
H8B3	0.5830	0.6488	0.5866	0.030*	
O1C	0.53025 (16)	0.53612 (10)	0.14682 (19)	0.0225 (4)	
H1C	0.5731	0.5436	0.2317	0.034*	
O2C	0.25159 (14)	0.24040 (9)	0.16008 (17)	0.0198 (3)	
O3C	0.15783 (15)	0.25965 (9)	−0.07388 (18)	0.0219 (4)	
C1C	0.4608 (2)	0.47356 (13)	0.1275 (3)	0.0176 (5)	
C2C	0.4694 (2)	0.42967 (13)	0.2407 (3)	0.0178 (5)	
H2C	0.5232	0.4436	0.3332	0.021*	
C3C	0.3977 (2)	0.36557 (14)	0.2147 (3)	0.0176 (5)	
H3C	0.4035	0.3362	0.2898	0.021*	
C4C	0.3163 (2)	0.34464 (13)	0.0758 (2)	0.0164 (5)	
C5C	0.3096 (2)	0.38827 (14)	−0.0364 (3)	0.0182 (5)	
H5C	0.2566	0.3741	−0.1290	0.022*	
C6C	0.3811 (2)	0.45228 (14)	−0.0109 (3)	0.0194 (5)	
H6C	0.3761	0.4812	−0.0861	0.023*	
C7C	0.2344 (2)	0.27824 (13)	0.0442 (2)	0.0173 (5)	
C8C	0.1753 (2)	0.17432 (14)	0.1380 (3)	0.0206 (5)	
H8C1	0.0942	0.1907	0.0882	0.031*	
H8C2	0.1926	0.1528	0.2278	0.031*	
H8C3	0.1885	0.1357	0.0835	0.031*	

### Atomic displacement parameters (Å^2^)

**Table:**

	*U*^11^	*U*^22^	*U*^33^	*U*^12^	*U*^13^	*U*^23^
O1A	0.0207 (9)	0.0252 (9)	0.0182 (10)	0.0078 (7)	0.0058 (8)	0.0009 (7)
O2A	0.0188 (9)	0.0202 (8)	0.0173 (9)	0.0021 (7)	0.0039 (7)	0.0009 (7)
O3A	0.0153 (8)	0.0200 (8)	0.0158 (8)	0.0043 (6)	0.0052 (7)	−0.0001 (7)
C1A	0.0133 (11)	0.0180 (11)	0.0177 (12)	0.0011 (9)	0.0058 (10)	0.0022 (9)
C2A	0.0203 (13)	0.0217 (11)	0.0146 (12)	0.0002 (10)	0.0054 (10)	−0.0029 (10)
C3A	0.0169 (12)	0.0206 (11)	0.0121 (11)	−0.0006 (9)	0.0033 (10)	0.0001 (9)
C4A	0.0123 (11)	0.0175 (11)	0.0165 (12)	−0.0020 (8)	0.0054 (9)	0.0009 (9)
C5A	0.0153 (12)	0.0176 (11)	0.0160 (12)	−0.0012 (9)	0.0068 (10)	−0.0019 (9)
C6A	0.0127 (11)	0.0226 (12)	0.0144 (12)	0.0001 (9)	0.0029 (10)	0.0007 (9)
C7A	0.0114 (11)	0.0176 (11)	0.0143 (12)	−0.0025 (8)	0.0047 (10)	−0.0002 (9)
C8A	0.0187 (12)	0.0170 (11)	0.0226 (13)	0.0026 (9)	0.0093 (10)	−0.0004 (10)
O1B	0.0213 (9)	0.0215 (9)	0.0171 (8)	0.0063 (7)	0.0053 (7)	0.0000 (7)
O2B	0.0197 (8)	0.0221 (8)	0.0141 (8)	0.0019 (7)	0.0020 (7)	0.0001 (7)
O3B	0.0194 (8)	0.0201 (8)	0.0147 (8)	0.0044 (6)	0.0056 (7)	0.0007 (7)
C1B	0.0141 (10)	0.0188 (11)	0.0177 (12)	−0.0030 (8)	0.0076 (9)	−0.0010 (9)
C2B	0.0173 (11)	0.0212 (11)	0.0136 (11)	−0.0008 (9)	0.0074 (9)	−0.0030 (9)
C3B	0.0133 (10)	0.0212 (11)	0.0139 (10)	−0.0015 (8)	0.0052 (9)	0.0003 (9)
C4B	0.0135 (10)	0.0143 (10)	0.0150 (11)	−0.0005 (8)	0.0055 (9)	0.0000 (8)
C5B	0.0150 (11)	0.0195 (11)	0.0133 (11)	0.0003 (8)	0.0053 (9)	−0.0003 (9)
C6B	0.0162 (11)	0.0190 (11)	0.0125 (10)	−0.0001 (9)	0.0054 (9)	0.0010 (9)
C7B	0.0150 (10)	0.0167 (10)	0.0154 (11)	−0.0017 (8)	0.0065 (9)	0.0001 (8)
C8B	0.0188 (10)	0.0197 (10)	0.0180 (10)	0.0059 (8)	0.0068 (8)	0.0016 (8)
O1C	0.0226 (9)	0.0218 (8)	0.0195 (9)	−0.0068 (7)	0.0075 (7)	−0.0003 (7)
O2C	0.0203 (8)	0.0195 (8)	0.0170 (8)	−0.0045 (6)	0.0070 (7)	0.0012 (7)
O3C	0.0200 (8)	0.0206 (8)	0.0166 (8)	−0.0012 (7)	0.0024 (7)	−0.0006 (7)
C1C	0.0135 (11)	0.0167 (11)	0.0213 (13)	0.0002 (8)	0.0076 (10)	0.0019 (9)
C2C	0.0168 (11)	0.0197 (11)	0.0149 (11)	0.0004 (8)	0.0061 (10)	−0.0015 (9)
C3C	0.0158 (11)	0.0211 (11)	0.0144 (12)	0.0001 (9)	0.0062 (9)	0.0011 (9)
C4C	0.0157 (11)	0.0148 (10)	0.0179 (12)	0.0010 (9)	0.0077 (9)	0.0010 (9)
C5C	0.0186 (11)	0.0199 (10)	0.0145 (11)	0.0013 (9)	0.0068 (9)	0.0001 (9)
C6C	0.0166 (12)	0.0213 (12)	0.0186 (12)	0.0009 (9)	0.0074 (10)	0.0046 (10)
C7C	0.0169 (11)	0.0155 (10)	0.0187 (12)	0.0026 (8)	0.0081 (10)	0.0008 (9)
C8C	0.0179 (13)	0.0199 (11)	0.0222 (13)	−0.0019 (9)	0.0084 (11)	0.0009 (10)

### Geometric parameters (Å, °)

**Table:**

O1A—C1A	1.357 (3)	C3B—H3B	0.93
O1A—H1A	0.82	C4B—C5B	1.403 (3)
O2A—C7A	1.218 (3)	C4B—C7B	1.472 (3)
O3A—C7A	1.340 (3)	C5B—C6B	1.389 (3)
O3A—C8A	1.446 (3)	C5B—H5B	0.93
C1A—C6A	1.387 (3)	C6B—H6B	0.93
C1A—C2A	1.397 (4)	C8B—H8B1	0.96
C2A—C3A	1.384 (3)	C8B—H8B2	0.96
C2A—H2A	0.93	C8B—H8B3	0.96
C3A—C4A	1.400 (3)	O1C—C1C	1.354 (3)
C3A—H3A	0.93	O1C—H1C	0.82
C4A—C5A	1.401 (4)	O2C—C7C	1.334 (3)
C4A—C7A	1.471 (3)	O2C—C8C	1.451 (3)
C5A—C6A	1.387 (3)	O3C—C7C	1.219 (3)
C5A—H5A	0.93	C1C—C6C	1.393 (3)
C6A—H6A	0.93	C1C—C2C	1.399 (3)
C8A—H8A1	0.96	C2C—C3C	1.383 (3)
C8A—H8A2	0.96	C2C—H2C	0.93
C8A—H8A3	0.96	C3C—C4C	1.400 (3)
O1B—C1B	1.359 (3)	C3C—H3C	0.93
O1B—H1B	0.82	C4C—C5C	1.398 (3)
O2B—C7B	1.221 (3)	C4C—C7C	1.484 (3)
O3B—C7B	1.331 (3)	C5C—C6C	1.380 (3)
O3B—C8B	1.449 (3)	C5C—H5C	0.93
C1B—C2B	1.388 (3)	C6C—H6C	0.93
C1B—C6B	1.399 (3)	C8C—H8C1	0.96
C2B—C3B	1.384 (3)	C8C—H8C2	0.96
C2B—H2B	0.93	C8C—H8C3	0.96
C3B—C4B	1.396 (3)		
			
C1A—O1A—H1A	109.5	C6B—C5B—H5B	119.7
C7A—O3A—C8A	116.4 (2)	C4B—C5B—H5B	119.7
O1A—C1A—C6A	122.9 (2)	C5B—C6B—C1B	119.4 (2)
O1A—C1A—C2A	117.1 (2)	C5B—C6B—H6B	120.3
C6A—C1A—C2A	120.1 (2)	C1B—C6B—H6B	120.3
C3A—C2A—C1A	119.4 (2)	O2B—C7B—O3B	121.7 (2)
C3A—C2A—H2A	120.3	O2B—C7B—C4B	124.7 (2)
C1A—C2A—H2A	120.3	O3B—C7B—C4B	113.54 (19)
C2A—C3A—C4A	121.0 (2)	O3B—C8B—H8B1	109.5
C2A—C3A—H3A	119.5	O3B—C8B—H8B2	109.5
C4A—C3A—H3A	119.5	H8B1—C8B—H8B2	109.5
C3A—C4A—C5A	119.0 (2)	O3B—C8B—H8B3	109.5
C3A—C4A—C7A	118.9 (2)	H8B1—C8B—H8B3	109.5
C5A—C4A—C7A	122.1 (2)	H8B2—C8B—H8B3	109.5
C6A—C5A—C4A	120.0 (2)	C1C—O1C—H1C	109.5
C6A—C5A—H5A	120.0	C7C—O2C—C8C	116.3 (2)
C4A—C5A—H5A	120.0	O1C—C1C—C6C	117.6 (2)
C5A—C6A—C1A	120.5 (2)	O1C—C1C—C2C	122.3 (2)
C5A—C6A—H6A	119.7	C6C—C1C—C2C	120.1 (2)
C1A—C6A—H6A	119.7	C3C—C2C—C1C	119.8 (2)
O2A—C7A—O3A	122.2 (2)	C3C—C2C—H2C	120.1
O2A—C7A—C4A	125.2 (2)	C1C—C2C—H2C	120.1
O3A—C7A—C4A	112.7 (2)	C2C—C3C—C4C	120.3 (2)
O3A—C8A—H8A1	109.5	C2C—C3C—H3C	119.8
O3A—C8A—H8A2	109.5	C4C—C3C—H3C	119.8
H8A1—C8A—H8A2	109.5	C5C—C4C—C3C	119.4 (2)
O3A—C8A—H8A3	109.5	C5C—C4C—C7C	118.8 (2)
H8A1—C8A—H8A3	109.5	C3C—C4C—C7C	121.8 (2)
H8A2—C8A—H8A3	109.5	C6C—C5C—C4C	120.4 (2)
C1B—O1B—H1B	109.5	C6C—C5C—H5C	119.8
C7B—O3B—C8B	116.7 (2)	C4C—C5C—H5C	119.8
O1B—C1B—C2B	117.3 (2)	C5C—C6C—C1C	119.9 (2)
O1B—C1B—C6B	122.2 (2)	C5C—C6C—H6C	120.0
C2B—C1B—C6B	120.5 (2)	C1C—C6C—H6C	120.0
C3B—C2B—C1B	119.7 (2)	O3C—C7C—O2C	122.4 (2)
C3B—C2B—H2B	120.1	O3C—C7C—C4C	124.7 (2)
C1B—C2B—H2B	120.1	O2C—C7C—C4C	112.8 (2)
C2B—C3B—C4B	121.0 (2)	O2C—C8C—H8C1	109.5
C2B—C3B—H3B	119.5	O2C—C8C—H8C2	109.5
C4B—C3B—H3B	119.5	H8C1—C8C—H8C2	109.5
C3B—C4B—C5B	118.8 (2)	O2C—C8C—H8C3	109.5
C3B—C4B—C7B	119.1 (2)	H8C1—C8C—H8C3	109.5
C5B—C4B—C7B	122.05 (19)	H8C2—C8C—H8C3	109.5
C6B—C5B—C4B	120.6 (2)		
			
O1A—C1A—C2A—C3A	180.0 (2)	O1B—C1B—C6B—C5B	179.4 (2)
C6A—C1A—C2A—C3A	1.2 (4)	C2B—C1B—C6B—C5B	1.0 (3)
C1A—C2A—C3A—C4A	−0.1 (4)	C8B—O3B—C7B—O2B	1.6 (3)
C2A—C3A—C4A—C5A	−1.2 (4)	C8B—O3B—C7B—C4B	−178.0 (2)
C2A—C3A—C4A—C7A	177.3 (2)	C3B—C4B—C7B—O2B	0.5 (3)
C3A—C4A—C5A—C6A	1.5 (3)	C5B—C4B—C7B—O2B	−178.1 (2)
C7A—C4A—C5A—C6A	−177.0 (2)	C3B—C4B—C7B—O3B	−179.9 (2)
C4A—C5A—C6A—C1A	−0.4 (3)	C5B—C4B—C7B—O3B	1.5 (3)
O1A—C1A—C6A—C5A	−179.7 (2)	O1C—C1C—C2C—C3C	−178.9 (2)
C2A—C1A—C6A—C5A	−1.0 (3)	C6C—C1C—C2C—C3C	−0.7 (3)
C8A—O3A—C7A—O2A	0.8 (3)	C1C—C2C—C3C—C4C	−0.3 (3)
C8A—O3A—C7A—C4A	−179.72 (19)	C2C—C3C—C4C—C5C	1.1 (3)
C3A—C4A—C7A—O2A	−3.4 (4)	C2C—C3C—C4C—C7C	−177.4 (2)
C5A—C4A—C7A—O2A	175.1 (2)	C3C—C4C—C5C—C6C	−1.1 (3)
C3A—C4A—C7A—O3A	177.1 (2)	C7C—C4C—C5C—C6C	177.5 (2)
C5A—C4A—C7A—O3A	−4.3 (3)	C4C—C5C—C6C—C1C	0.2 (3)
O1B—C1B—C2B—C3B	179.9 (2)	O1C—C1C—C6C—C5C	179.0 (2)
C6B—C1B—C2B—C3B	−1.6 (3)	C2C—C1C—C6C—C5C	0.7 (3)
C1B—C2B—C3B—C4B	0.8 (4)	C8C—O2C—C7C—O3C	1.2 (3)
C2B—C3B—C4B—C5B	0.5 (3)	C8C—O2C—C7C—C4C	−179.69 (18)
C2B—C3B—C4B—C7B	−178.1 (2)	C5C—C4C—C7C—O3C	−2.4 (3)
C3B—C4B—C5B—C6B	−1.2 (3)	C3C—C4C—C7C—O3C	176.2 (2)
C7B—C4B—C5B—C6B	177.5 (2)	C5C—C4C—C7C—O2C	178.6 (2)
C4B—C5B—C6B—C1B	0.4 (3)	C3C—C4C—C7C—O2C	−2.9 (3)

### Hydrogen-bond geometry (Å, °)

**Table:**

*D*—H···*A*	*D*—H	H···*A*	*D*···*A*	*D*—H···*A*
O1A—H1A···O2A^i^	0.82	1.96	2.770 (2)	168
O1B—H1B···O3C^ii^	0.82	1.93	2.729 (2)	167
O1C—H1C···O2B	0.82	1.92	2.729 (2)	167
C6A—H6A···O2C	0.93	2.58	3.343 (3)	140
C8C—H8C1···Cg1^i^	0.96	2.76	3.539 (3)	139
C8C—H8C3···Cg2	0.96	2.70	3.442 (3)	134
C8A—H8A1···Cg3^iii^	0.96	2.68	3.515 (3)	145
C8B—H8B3···Cg3^iv^	0.96	2.78	3.655 (4)	151

Symmetry codes: (i) *x*−1/2, −*y*+1/2, *z*−1/2; (ii) *x*+1, *y*, *z*+1; (iii) *x*+1/2, −*y*+1/2, *z*+1/2; (iv) *x*−1/2, *y*−1/2, *z*.
